# Cyclin A1 (CCNA1) inhibits osteoporosis by suppressing transforming growth factor-beta (TGF-beta) pathway in osteoblasts

**DOI:** 10.1186/s12891-024-07303-6

**Published:** 2024-03-07

**Authors:** Xiao Du, Chuanyi Zang, Qinglei Wang

**Affiliations:** Department of Orthopedics, Beijing Geriatric Hospital, No.118 Hot Spring Road, Haidian District 100095, Beijing, China

**Keywords:** Osteoporosis, Osteogenesis, CCNA1, TGF-beta, SMAD, Dexamethasone

## Abstract

**Background:**

Osteoporosis is a genetic disease caused by the imbalance between osteoblast-led bone formation and osteoclast-induced bone resorption. However, further gene-related pathogenesis remains to be elucidated.

**Methods:**

The aberrant expressed genes in osteoporosis was identified by analyzing the microarray profile GSE100609. Serum samples of patients with osteoporosis and normal group were collected, and the mRNA expression of candidate genes was detected by quantitative real-time polymerase chain reaction (qRT-PCR). The mouse cranial osteoblast MC3T3-E1 cells were treated with dexamethasone (DEX) to mimic osteoporosis in vitro. Alizarin Red staining and alkaline phosphatase (ALP) staining methods were combined to measure matrix mineralization deposition of MC3T3-E1 cells. Meanwhile, the expression of osteogenesis related genes including alkaline phosphatase (ALP), osteocalcin (OCN), osteopontin (OPN), Osterix, and bone morphogenetic protein 2 (BMP2) were evaluated by qRT-PCR and western blotting methods. Then the effects of candidate genes on regulating impede bone loss caused by ovariectomy (OVX) in mice were studied.

**Results:**

Cyclin A1 (CCNA1) was found to be significantly upregulated in serum of osteoporosis patients and the osteoporosis model cells, which was in line with the bioinformatic analysis. The osteogenic differentiation ability of MC3T3-E1 cells was inhibited by DEX treatment, which was manifested by decreased Alizarin Red staining intensity, ALP staining intensity, and expression levels of ALP, OCN, OPN, Osterix, and BMP2. The effects of CCNA1 inhibition on regulating osteogenesis were opposite to that of DEX. Then, Kyoto Encyclopedia of Genes and Genomes (KEGG) analysis demonstrated that genes negatively associated with CCNA1 were enriched in the TGF-beta signaling pathway. Inhibitor of TGF-beta signaling pathway partly reversed osteogenesis induced by suppressed CCNA1. Furthermore, suppressed CCNA1 relieved bone mass of OVX mice in vivo.

**Conclusion:**

Downregulation of CCNA1 could activate TGF-beta signaling pathway and promote bone formation, thus playing a role in treatment of osteoporosis.

**Supplementary Information:**

The online version contains supplementary material available at 10.1186/s12891-024-07303-6.

## Introduction

Osteoporosis is a common bone metabolic disease, characterized by bone strength and bone mineral density (BMD) decrease, leading to chronic pain, disability and, in severe cases, life threatening [1, 2]. Previous studies have shown that osteoporosis is more common in postmenopausal women and increases the risk of fractures [3]. Osteogenic differentiation is a key factor in bone regeneration, it is significant to clarify the regulatory mechanism of osteogenic differentiation and calcification for improving the treatment of osteoporosis [4]. A better understanding of the molecular mechanisms that participate in osteogenesis may help to treat osteoporosis. BMD changes are a major risk factor for osteoporosis, and more than 60% of the variation in BMD is due to genetic factors [5]. Therefore, identifying new therapeutic targets and biomarkers associated with osteoporosis has clinical significance.

Osteogenesis is a complex dynamic gene-modified program of osteoblasts that leads to the production of a collagenous mineralized matrix, playing a crucial role in bone homeostasis [6]. Bone formation controlled by osteoblasts involves multiple genetic and epigenetic regulation mechanisms [7]. Cyclin A1 (CCNA1) protein is a member of cyclin family, and is a positive regulator of cell cycle [8]. In addition, CCNA1 is involved in the occurrence, development, invasion and metastasis of a variety of tumors [9, 10]. Earlier bioinformatics analysis results demonstrated that CCNA1 was aberrant expressed gene in osteoporosis. Therefore, we aims to seek for the function of CCNA1 and the underlying mechanisms in osteoporosis, thereby to open up new ideas for the treatment of osteoporosis and other bone metabolism-related diseases.

## Materials and methods

### Microarray analysis

A microarray profile GSE100609 related to osteoporosis was downloaded from the Gene Expression Omnibus (GEO) database. All differentially expressed genes (DEGs) of osteoporotic group (*n* = 3) and non-osteoporotic group (*n* = 3) were identified and then screened under the standard od *P* < 0.05 and |log_2_FC| ≥ 1.5. After that, pearson correlation analysis was performed between DEGs, and the Kyoto Encyclopedia of Genes and Genomes (KEGG) analysis was applied to analyze the related-pathways of enriched genes.

### Patients

Approved by the Ethics Committee of Beijing Geriatric Hospital, 20 patients with osteoporosis diagnosed in Beijing Geriatric Hospital and 20 non-osteoporosis controls who volunteered to participate in this study signed informed consent. Then, 5 mL Fasting blood was collected from all subjects, and the isolated serum samples were stored at minus 80 ℃ for subsequent experiments.

### Osteoporosis model cell establishment

The mouse cranial osteoblast MC3T3-E1 cells were obtained from Shanghai Institute of Biochemistry and Cell Biology (Shanghai, China), and cultured in Dulbecco’s modified Eagle medium (Hyclone, Logan, USA) containing 10% fetal bovine serum (FBS, Gibco, Carlsbad, USA), 100 mg/mL streptomycin, and 100 U/mL penicillin (Solarbio, Beijing, China).

For osteoporosis model cell establishment, MC3T3-E1 cells at a density of 10^4^ cells per well were seeded into 6-well plates and cultured until the cells reached to over 60% confluence. Then 100 µM dexamethasone (DEX; Sigma-Aldrich; Merck, USA) [11], 10 mM beta-glycerophosphate (Sigma-Aldrich; Merck, USA) and 50 µg/mL ascorbic acid Sigma-Aldrich; Merck, USA) [12, 13] were added into the medium, and replenished every 3 days.

### Alizarin red staining

Alizarin Red staining was applied to measure matrix mineralization deposition in MC3T3-E1 cells [14]. In brief, DEX-induced osteoporosis cells were fixed in 4% paraformaldehyde (aladdin, Shanghai, China) for 10 min, and then washed with distilled water and stained with 1% Alizarin Red in accordance with the manufacturer’s protocol (Leagene, Beijing, China). Finally, the stained cells were rinsed with distilled water, matrix calcification was observed, and red deposition were observed under the microscope (Nikon, Tokyo, Japan).

### Alkaline phosphatase (ALP) staining

For ALP staining, the MC3T3-E1 cells were fixed in 70% ethanol for 1 h, and then washed with distilled water 3 times. ALP staining was performed with an ALP staining kit (Sidansai, Shanghai, China) according to the manufacturer’s protocol. The stained cells were then photographed with a microscope (Zeiss, Oberkochen, Germany). Then the stain cells were monitored at 562 nm (BioTek, Winooski, USA) to quantify ALP staining intensity.

### Cell transfection

Exponential DEX-induced osteoporosis cells were collected for transfection. Two kinds of si-CCNA1 plasmids (1# 5’-CAGCTACACCAAGATTTCTTGGAAA-3’, 2# 5’-CATACCATGTGAGAGCACTTCTGTA-3’) and negative control (si-NC, 5’-TTCTCCGAACGTGTACGTTT-3’) have been designed by GenePharma Company (Shanghai, China). Transfection was performed using Lipofectamine 3000 transfection kits (Invitrogen, Carlsbad, USA). Aftern transfection triumphantly, the expression of *CCNA1* of DEX-induced osteoporosis cells was detected by quantitative real-time polymerase chain reaction (qRT-PCR).

### qRT-PCR

qRT-PCR was applied to evaluate levels of CCNA1, alkaline phosphatase (ALP), osteocalcin (OCN), osteopontin (OPN), Osterix, and bone morphogenetic protein 2 (BMP2), par-6 family cell polarity regulator alpha (PARD6A) and interferon beta 1 (IFNB1). Firstly, total RNA of DEX-induced osteoporosis cells were extracted by Trizol RNA isolation reagent (Invitrogen, Carlsbad, USA). Agilent RNA 6000 Nano Kit and Agilent 2100 Bioanalyzer (Agilent, Santa Clara, CA) were applied to detect RNA integrity and concentration. Then, qRT-PCR was performed on an ABI 7500 quantitative polymerase chain reaction system (Life Technologies, Carlsbad, USA). The relative expressions of genes were calculated using the 2^−ΔΔCt^ method. Primer sequences were as followed:

CCNA1: forward, 5′-TGAAGTAGACACCGGCACAC-3′;

reverse, 5′-CACTCCTTGTCGCCTCAAGT-3′,

ALP: forward, 5′-CCAACTCTTTTGTGCCAGAGA-3′;

reverse, 5′-GGCTACATTGGTGTTGAGCTTTT-3′,

OCN: forward, 5′-CTGACCTCACAGATCCCAAGC-3′;

reverse, 5′-TGGTCTGATAGCTCGTCACAAG-3′,

OPN: forward, 5′-AATACCCAGATGCTGTGGCC-3′;

reverse, 5′-ACGGCTGTCCCAATCAGAAG-3′,

Osterix: forward, 5′-GAAGCGACCACTTGAGCACAT-3′;

reverse, 5′-TGTCCAAACTCATCAATGTATCT-3′,

BMP2: forward, 5′-ACCCGCTGTCTTCTAGCGT-3′;

reverse, 5′-TTTCAGGCCGAACATGCTGAG-3′,

PARD6A: forward, 5′-GCGGGTTCCAGGAATCTTCA-3′;

reverse, 5′-CACTGTGAAGTCCCTGCCAT-3′,

IFNB1: forward, 5′-TGCTCTGGCACAACAGGTAG-3′;

reverse, 5′-TATGGTCCAGGCACAGTGAC-3′,

GAPDH: forward: 5′-CGAGCCACATCGCTCAGACA-3′;

reverse: 5′-GTGGTGAAGACGCCAGTGGA-3′).

### Western blotting

DEX-induced osteoporosis cells were lysed to obtain total protein by RIPA reagent (Sigma, NJ, USA). After quantified by a BCA protein assay (Thermol Fisher, CA, USA), protein was isolated by 10% SDS-PAGE and transferred to polyvinylidene fluoride membranes (Millipore, MA, USA). After being blocked with 5% skim milk, the membranes were incubated with primary antibodies purchased from abcam (Cambridge, UK) for anti-ALP (1:500, ALP), anti-OCN (1:1000, ab309521), anti-OPN (1:1000, ab214050), anti-Sp7/Osterix (1:1000, ab227820), anti-BMP2 (1:1000, ab214821), anti-SMAD2 (1:2000, ab40855), anti-SMAD3 (1:2000, ab40854), anti-p-SMAD2 (1:1000, ab280888), and anti-p-SMAD3 (1:2000, ab52903), and GADPH (1:1000, ab8245) overnight at 4℃. Then membranes were washed with TBST and incubated with secondary antibody (1:5000, ab6721). Protein bands were visualized using a gel imaging system (Bio-Rad, Hercules, USA).

### Ovariectomized (OVX)-induced osteoporosis mouse model establishment

The sh-CCNA1 plasmids (5’-GCACGAGAATTGAGAATTAGA-3’) and negative control (sh-NC, 5’-GCCCAATTGTGCAGTGTGAAA-3’) were designed by GenePharma Company (Shanghai, China). Animal use was approved by the Ethics Committee of Beijing Geriatric Hospital. Twelve-week-old C57BL/6 female mice were obtained from Animal Center of Academy of Military Medical Sciences. The animal experiments were conducted under the Guide for the Care and Use of Laboratory Animals and were carried out following the institutional ethical principles for animal experiments. Under pathogen-free conditions, mice were housed (4–5/cage) at 25℃ on a 12-h light/dark cycle, with adaptive feeding for 2 weeks. As previously reported, OVX-induced osteoporosis mouse model was established [15]. After intraperitoneal injection of ketamine (100 mg/kg) and xylazine (10 mg/kg), mice were performed with bilateral ovariectomy by dorsal approach. Mice in the sham group were subjected to incision and ovary exposure, but not to excision of the ovary. After four weeks of operation, 100 µL sh-CCNA1 or sh-NC was injected subcutaneously once a week for another four weeks. All mice were euthanized by intraperitoneal injection of pentobarbital sodium 160 mg/kg, and the femurs were removed after the last injection.

### Micro-computed tomography

A Scanco vivaCT 40 (Scanco Medical AG, Bassersdorf, Switzerland) was employed for micro-computed tomography (µCT) as previously reported27. Using 6-µm pixel size, we set an X-ray source at 60 kV and scanned the excised left distal metaphysis of the femurs. We focused on a ~ 0.5 mm proximal region in the most distal part of the growth plate.

The femurs of mice were fixed with 4% paraformaldehyde for 24 h and then scanned by a Scanco vivaCT 40 (Scanco Medical AG, Bassersdorf, Switzerland). An X-ray source at 60 kV was set, the femurs were then scanned. Several bone-related parameters were analyzed, including the bone volume/total volume (BV/TV, %), trabecular thickness (Tb.Th, µm), trabecular number (Tb.N, mm^− 1^), trabecular separation (Tb.Sp, µm), and BMD (g/cm^3^).

### Histology of bone tissues

Following Micro-computed tomography analysis, femurs were decalcified in 14% EDTA (Sigma-Aldrich, Sydney, NSW, Australia) at 37℃ for a week, and then embedded into paraffin for sectioning. Hematoxylin and eosin (H&E) staining was performed. Section images were acquired under a microscope Olympus FV1000 (Tokyo, Japan).

### Statistical analysis

Statistical analyses were performed using the SPSS version 17.0 (SPSS Inc., Chicago, USA). All data were expressed as mean ± SD. Comparison between two groups was determined by student’s t-test. One-way ANOVA was used for comparison among different groups. *p* values less than 0.05 were considered to be significantly different.

## Results

### CCNA1 expression is significantly upregulated in osteoporosis

**Fig. 1:**
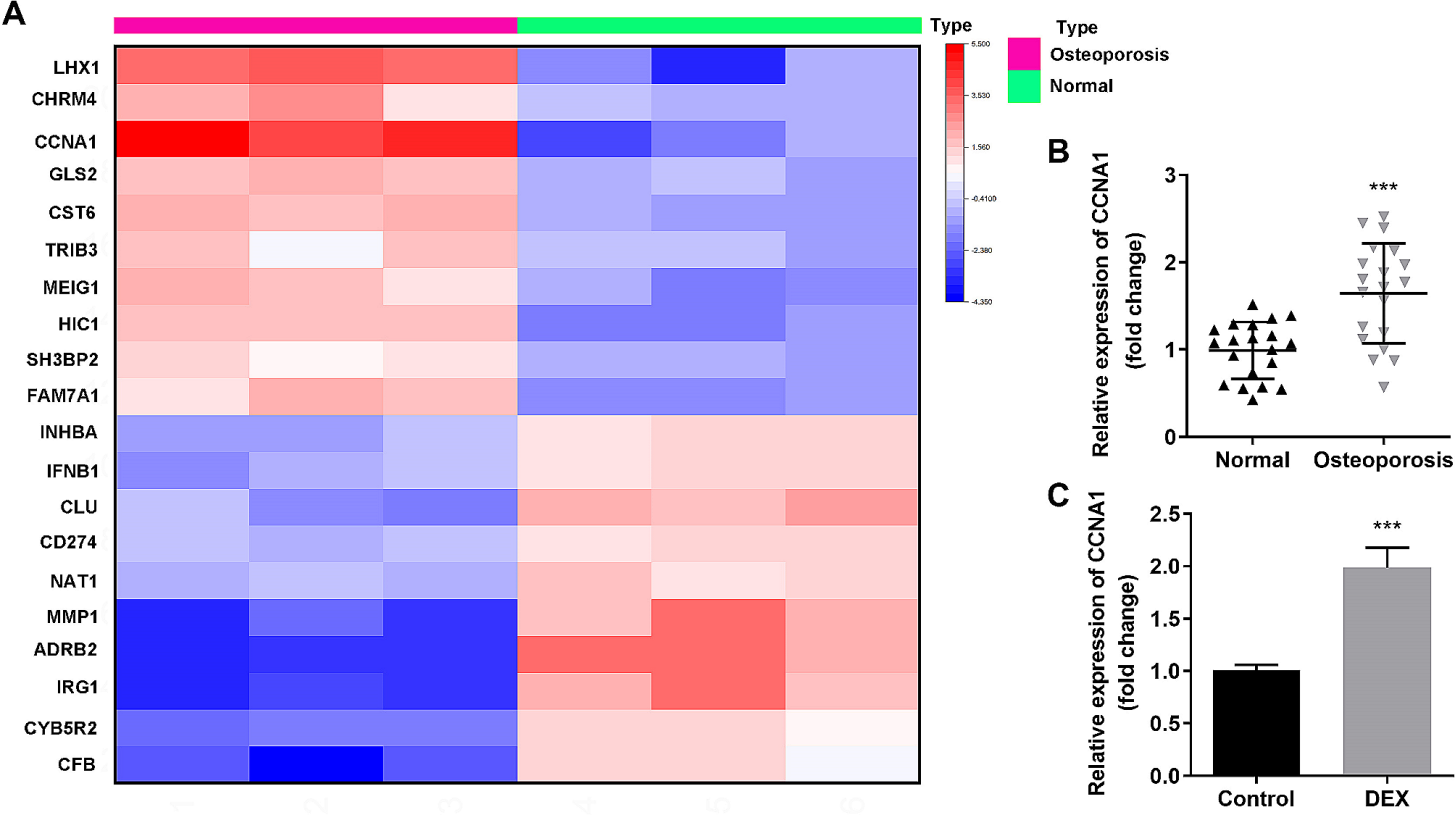
Dramatic upregulation of CCNA1 in osteoporosis. **(A)** Heat map of DEGs identified in patients with osteoporosis. **(B)** Detection of CCNA1 expression in patients with osteoporosis compared with that in healthy volunteers using qRT-PCR. **(C)** Detection of CCNA1 expression in MC3T3-E1 cells before and after DEX treatment using qRT-PCR. ***p < 0.001

Firstly, microarray analysis of GSE100609 related to osteoporosis was conducted to identify genes which are aberrant expressed. As shown in Figs. 1A and 20 DEGs were identified in patients with osteoporosis, among which CCNA1 was markedly overexpressed. Additionally, the mRNA expression of CCNA1 was upregulated in the serum of patients with osteoporosis compared with the normal group (Fig. 1B). Moreover, the CCNA1 expression in DEX-treated MC3T3-E1 cells was significantly higher than that in untreated group (Fig. 1 C).

### DEX-treated MC3T3-E1 cells were successfully established as osteoporosis model cells

**Fig. 2:**
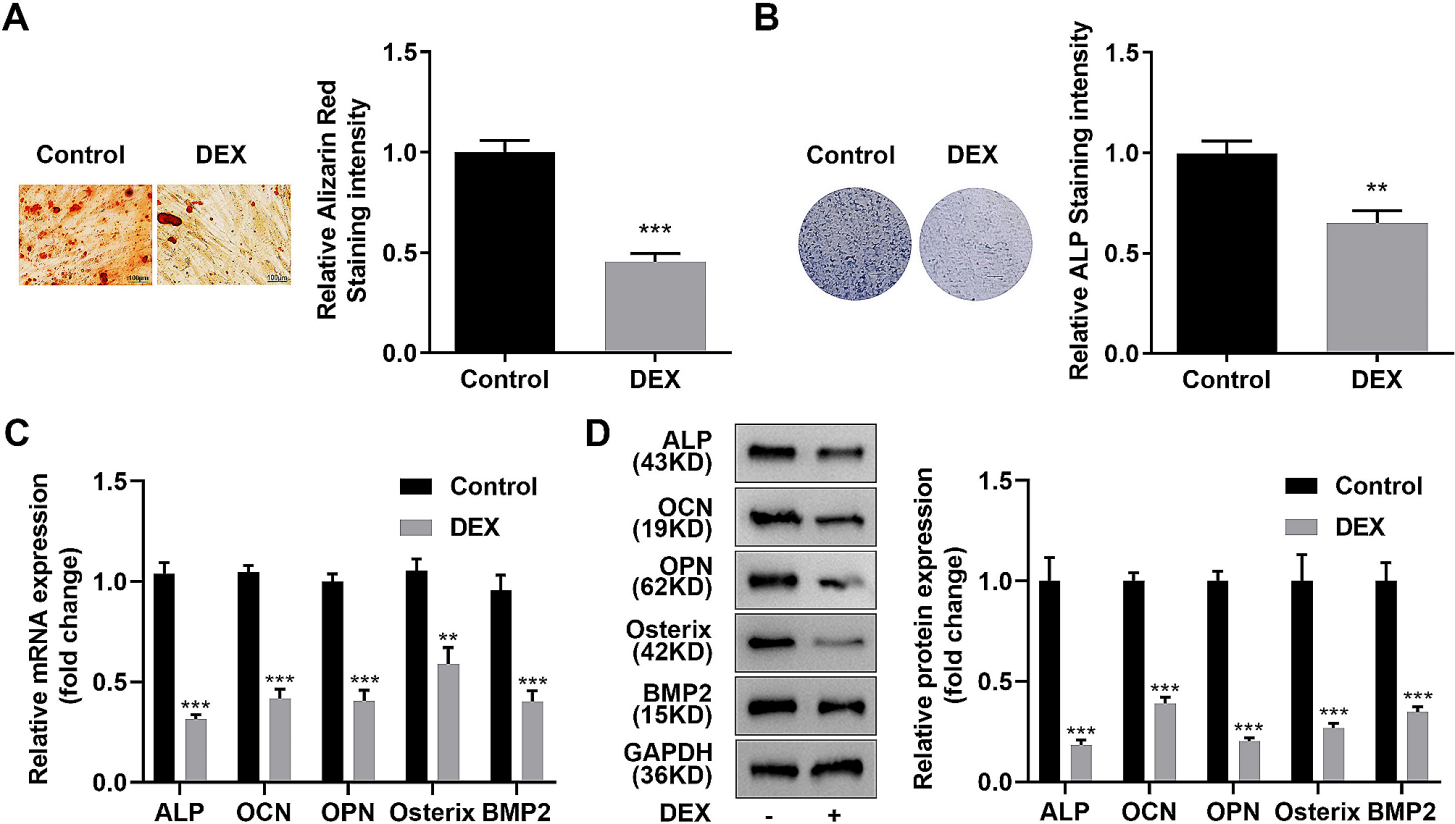
Successful establishment of DEX-treated MC3T3-E1 cells as osteoporosis model cells. **(A)** Image and quantification of Alizarin Red stained DEX-treated MC3T3-E1 cells. **(B)** Image and quantification of ALP stained DEX-treated MC3T3-E1 cells. **C-D**. Measurement of the expression levels of ALP, OCN, OPN, osterix, and BMP2 using qRT-PCR and western blotting. **p < 0.01, ***p < 0.001

Then osteogenesis of MC3T3-E1 cells of each group was evaluated to verify the success establishment of osteoporosis model cells. After treated with DEX, number of Alizarin Red stained cells and ALP stained cells were significantly decreased in MC3T3-E1 cells (Fig. 2A and B). Meanwhile, the expression of osteogenic-related proteins such as ALP, OCN, OPN, Osterix, and BMP2 were dramatically downregulated in DEX-treated MC3T3-E1 cells (Fig. 2C and D). These results suggested that DEX treatment inhibited osteogenesis of MC3T3-E1 cells, indicating that DEX-induced osteoporosis model cells were successfully established.

### Knockdown CCNA1 promoted osteogenesis of MC3T3-E1 cells

**Fig. 3:**
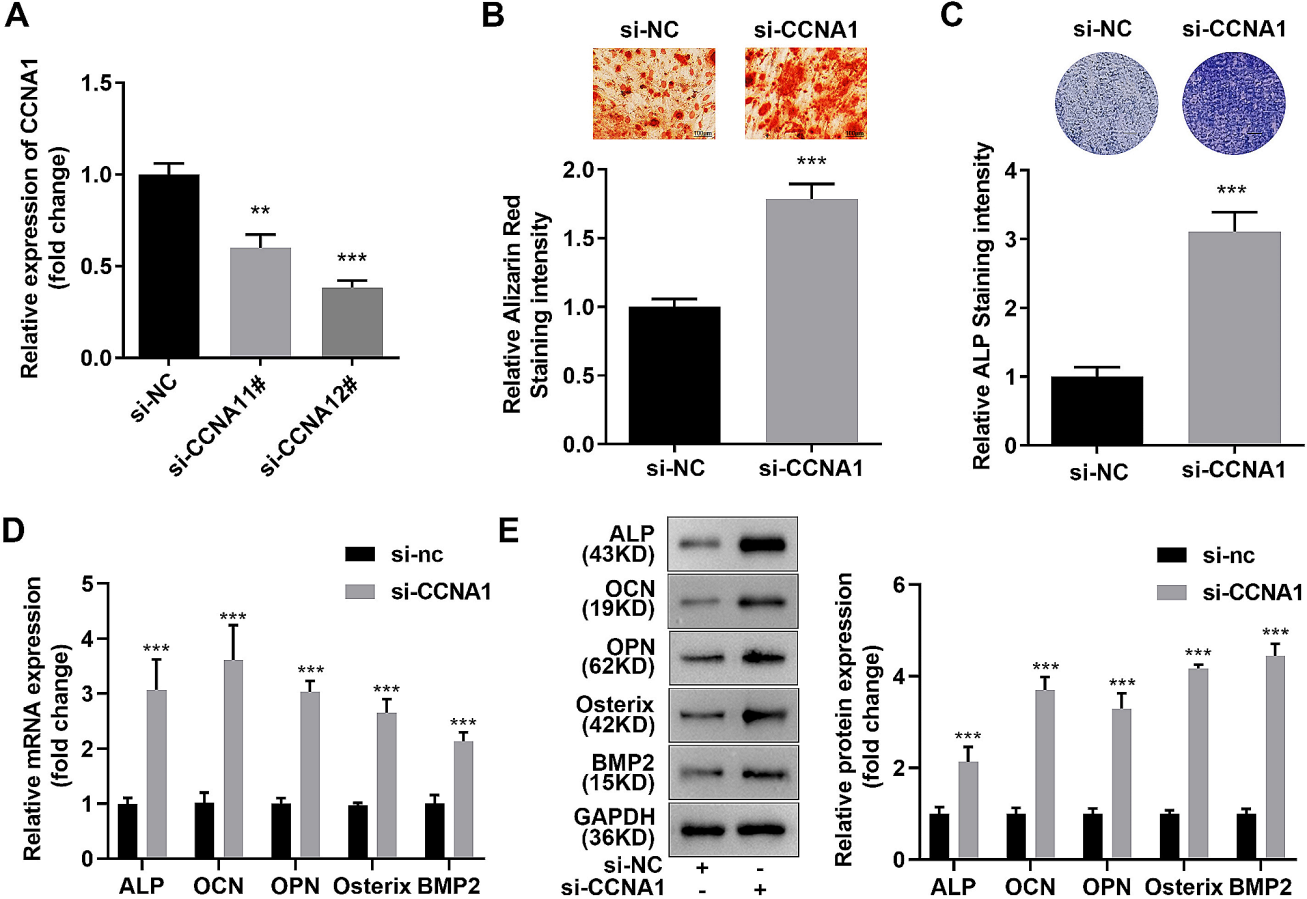
CCNA1 knockdown promotes osteogenesis of MC3T3-E1 cells. **(A)** Detection of CCNA1 expression levels by qRT-PCR after transfection. **(B)** Image and quantification of Alizarin Red stained MC3T3-E1 cells with inhibited CCNA1. **(C)** Image and quantification of ALP stained MC3T3-E1 cells with inhibited CCNA1. **D-E**. Expression levels of ALP, OCN, OPN, Osterix, and BMP2 measured by qRT-PCR and western blotting. **p < 0.01, ***p < 0.001

Afterwards, the role of CCNA1 in modulating osteogenesis of MC3T3-E1 cells was studied. As indicated in Fig. 3A, CCNA1 expression was more remarkably downregulated after transfected with si-CCNA1 1# plasmid. After CCNA1 was suppressed, number of Alizarin Red stained cells and ALP stained cells were significantly increased (Fig. 3B C). Meanwhile, the expression of osteogenic-related proteins such as ALP, OCN, OPN, Osterix, and BMP2 were dramatically upregulated in MC3T3-E1 cells (Fig. 3D and E).

### CCNA1 expression was negatively related with TGF-beta signaling pathway

**Fig. 4:**
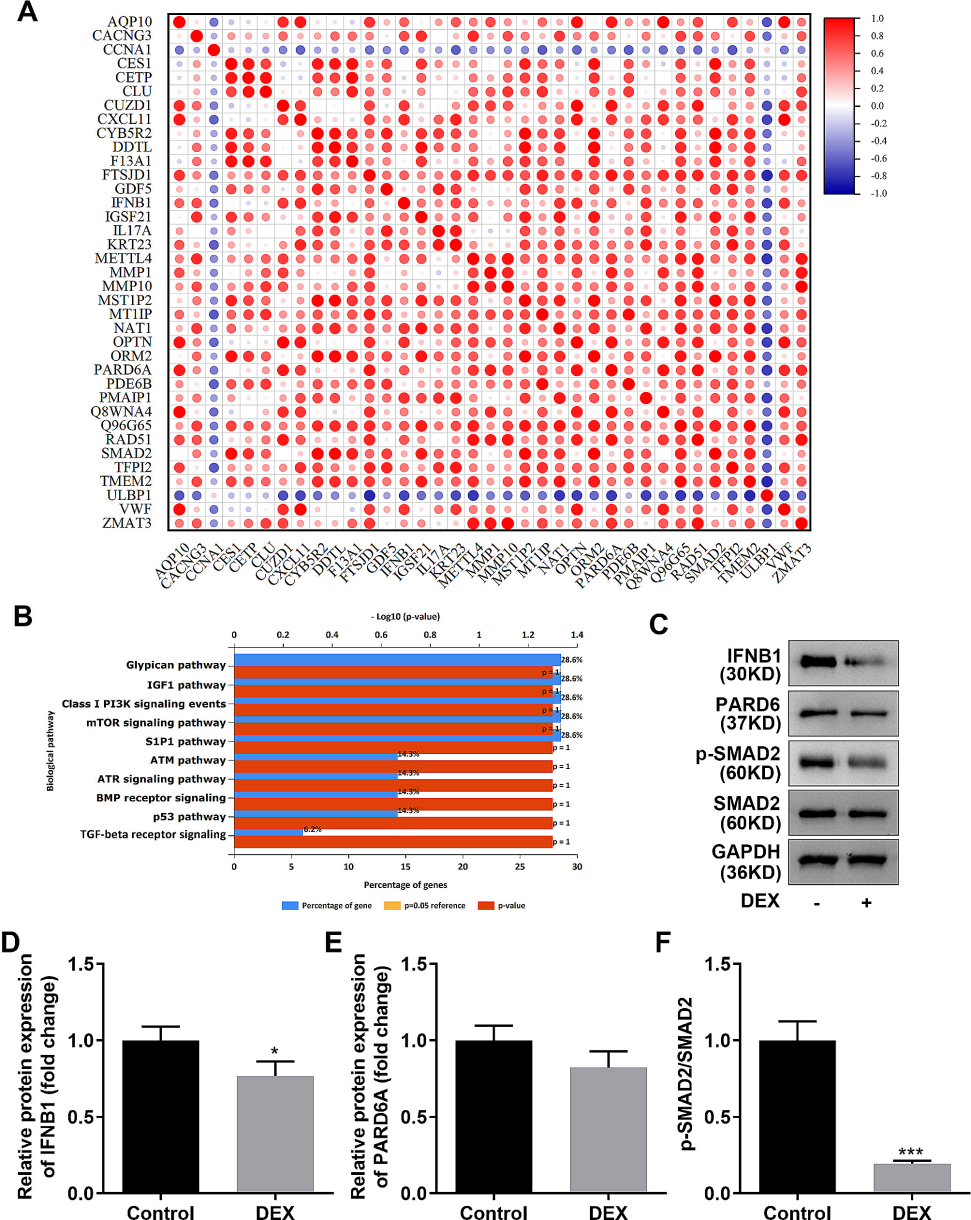
CCNA1 expression is negatively related to the TGF-beta signaling pathway. **(A)** Pearson correlation analysis between CCNA1 and its negatively related genes. **(B)** KEGG analysis of genes inversely associated with CCNA1 gene. **C-F**. Image and quantification of expression levels of PARD6A and IFNB1 enriched in TGF-beta signaling pathway accessed by western blotting. *p < 0.05, ***p < 0.001

Next, pearson correlation analysis and KEGG analysis demonstrated that genes negatively related to CCNA1 were enriched in 10 pathways, including the glypican, IGF1, PI3K, mTOR, S1P1, ATM, ATR, BMP receptor, p53, and TGF-beta receptor signaling pathways (Fig. 4A and B). Interestingly, the expression of PARD6A and p-SMAD2, two genes enriched in the TGF-beta receptor signaling pathway, were significantly decreased in DEX-induced osteoporosis cells compared with that in control cells (Fig. 4C-4 F).

### Suppressed CCNA1 promoted osteogenesis by activating TGF-beta signaling pathway

**Fig. 5:**
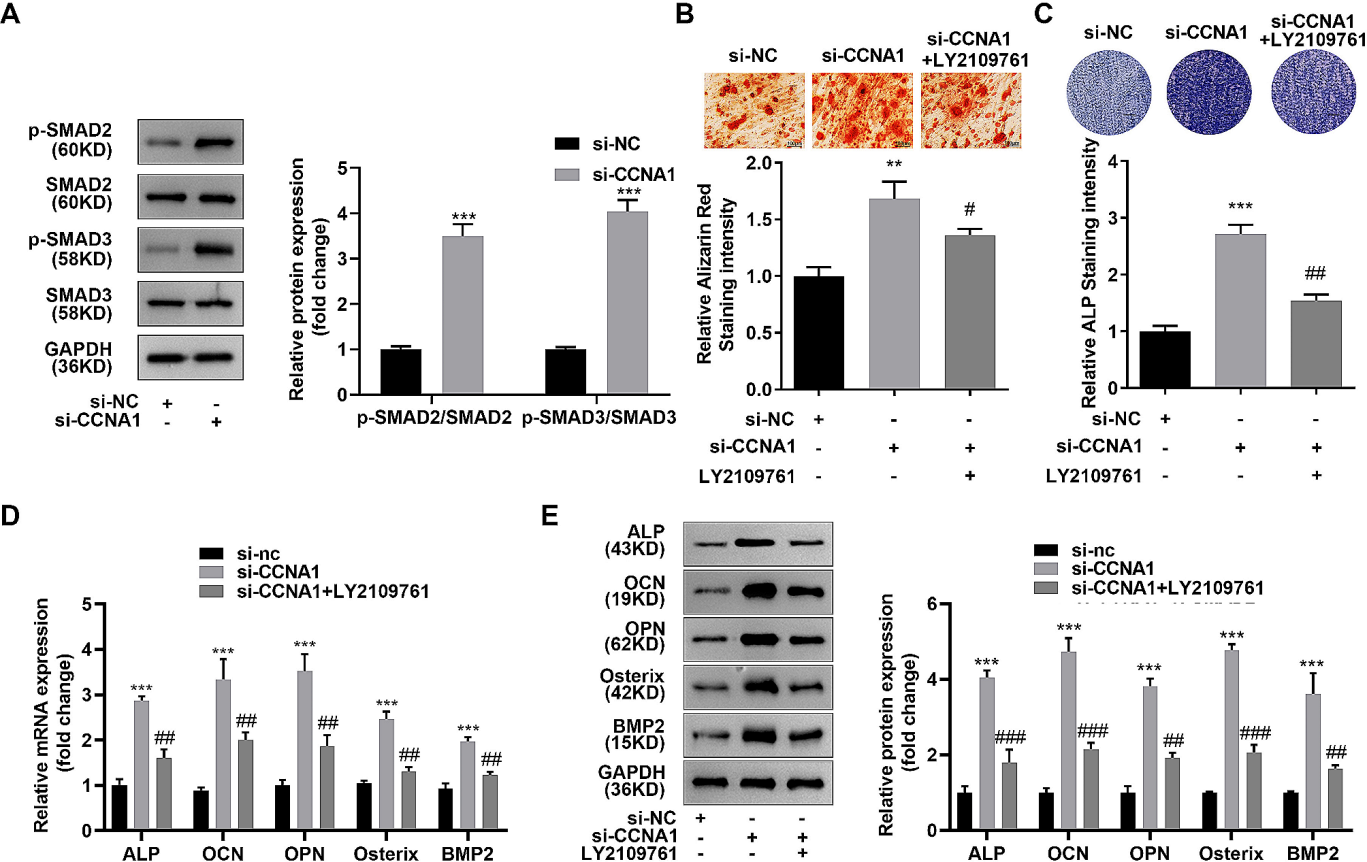
Suppressed CCNA1 promoted osteogenesis by activating TGF-beta signaling pathway. **(A)** Protein expression of SMAD2/3 as well as phosphorylated SMAD2/3 measured by western blotting. **(B)** Image and quantification of Alizarin Red stained MC3T3-E1 cells after LY2109761 treatment. **(C)** Image and quantification of ALP stained cells MC3T3-E1 cells after LY2109761 treatment. **D-E.** Expression levels of ALP, OCN, OPN, Osterix, and BMP2 measured by qRT-PCR and western blotting after LY2109761 treatment. **p < 0.01, ***p < 0.001, compared with si-nc group. #*p* < 0.05, ##*p* < 0.01, ###*p* < 0.001, compared with si-CCNA1 group

Then, expression levels of SMAD2/3 and phosphorylated SMAD2/3 were notably increased induced by silenced CCNA1 in DEX-induced osteoporosis cells (Fig. 5A). Furthermore, LY2109761, an inhibitor of the TGF-beta signaling pathway, suppressed the intensity of Alizarin Red and ALP intensity (Fig. 5B C). Meanwhile, the expression levels of ALP, OCN, OPN, osterix, and BMP2 increased by si-CCNA1 were also downregulated by LY2109761 (Fig. 5D and E).

### Suppressed CCNA1 relieved bone mass in vivo

**Fig. 6:**
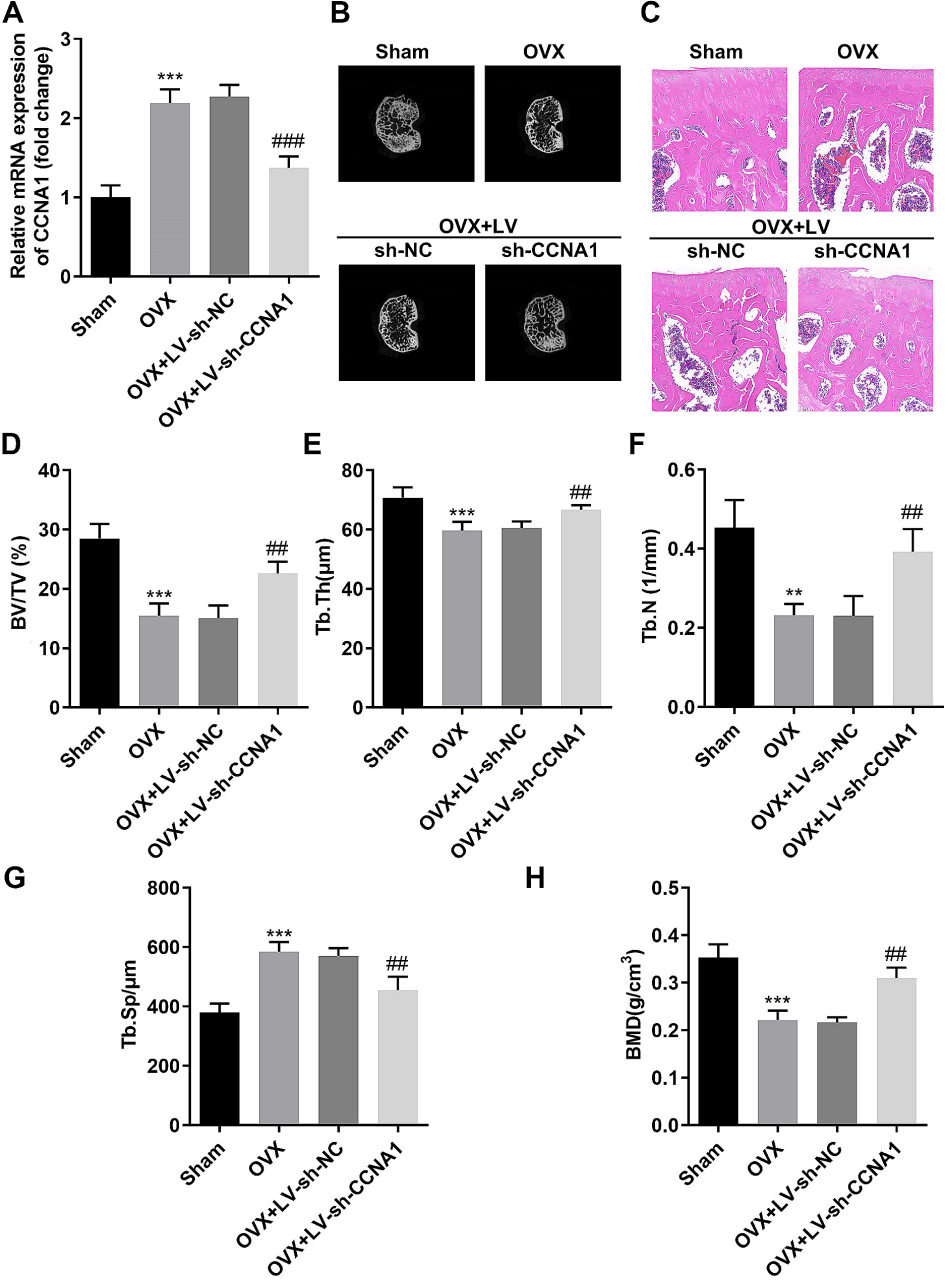
Suppressed CCNA1 relieves bone mass in vivo. **(A)** Detection of CCNA1 expression levels by qRT-PCR in bone tissues of all mice. **(B)** Representative µCT images were photoed to showthe bone loss. **(C)** Representative images of HE staining of decalcified bone sections. **D-H.** Quantification of micro-computed tomography data: BV/TV, Tb. Th, Tb. N, Tb.Sp. and BMD. **p < 0.01, ***p < 0.001, compared with Sham group. ##*p* < 0.01, compared with OVX + LV-sh-NC group

Afterwards, CCNA1 was successfully downregulated in bone tissues of OVX mice injected with LV-sh-CCNA1 (Fig. 6A). The right tibials of mice were analyzed to perform the micro-computed tomography., and the results demonstrated that inhibition of CCNA1 prevented the extensive bone loss induced by the OVX procedure in mice femurs (Fig. 6B). Consistently, histological examination showed that the bone volume and bone surface were both well maintained in the CCNA1 inhibition group compared with the OVX mice (Fig. 6C). The quantitative analysis revealed that differences in BV/TV, Tb.Th, Tb.N, and Tb.Sp in trabecular bone were all significant between sham and OVX groups (Fig. 6D and G). Moreover, BMD was also dramatically suppressed by OVX surgery (Fig. 6H). These data revealed that OVX surgery prominentlyattenuated bone microstructure and bone mass; however, knockdown of CCNA1 dramatically reversed the effects of OVX treatment.

## Discussion

Osteoporosis is the result of the disruption of the dynamic balance between bone formation and bone resorption [16, 17]. During this process, osteoblasts play a key role in bone formation and are responsible for osteogenesis and bone regeneration [18]. In addition, inhibition of osteogenic differentiation can cause bone defects after trauma and seriously affect the quality of life [19]. Therefore, studying of the mechanism of osteogenic differentiation is of great significance for the treatment of diseases, such as heterotopic mineralization and osteoporosis.

Studies have shown that osteogenic differentiation can be evaluated using various bone metabolism markers [20, 21]. For instance, the intensity of ALP and ARS is generally used to detect early and late mineralization states, which indicate bone formation by osteoblasts [22]. Additionally, OPN (regulates biomineralization in bone tissue and aids in growth reduction and accumulation of calcium crystals in epithelial tissue), osterix (bone formation-related genes), and BMP2 (stimulates bone mineralization and osteoblast differentiation) have been reported to be related to bone metabolism regulators [23–25]. Moreover, elevated expression of bone turnover markers including ALP and OCN has also been reported to be related to the remission of osteoporosis [26]. In this study, we evaluated osteogenesis by detecting mineralization levels, ALP, OCN, OPN, osterix, and BMP2 expression in osteoblasts.

Genetic association research is an effective method to explore the molecular genetic mechanisms of many complex diseases including osteoporosis in recent years [27, 28]. Recent genome-wide association studies meta-analyses and large sample association studies have shown that many genes are associated with osteoporosis and fracture risk [29–31]. An increasing number of abnormally expressed genes have been identified as the key regulators of osteoblast differentiation [32, 33]. The abnormal expression of genes involved in the differentiation of stem cells into osteoblasts and the potential role of stem cells in osteogenesis have been reported [34, 35]; however, their relative functional significance is not fully understood. SOST gene is a candidate gene for osteoporosis [36]. Inhibition of SOST and its products induces osteoblastic activation and promotes bone formation [37]. Likewise, the knockdown of genes involved in bone metabolism, such as RANKL, RANK, and DKK-1, provides promising therapies based on genetic engineering [38–40]. Hence, there is an urgent need to improve the gene network related to bone metabolism for osteoporosis treatment. Our data suggested that CCNA1 screened from the bioinformatics analysis was dramatically overexpressed in the serum of patients with osteoporosis, indicating that CCNA1 may be a potential gene related to bone metabolism. Therefore, the mechanisms of action of CCNA1 were investigated in this study.

Dexamethasone (DEX) is an artificial compound of long-acting glucocorticoid. Glucocorticoid induced osteoporosis (GIOP) is a kind of thin-bone hormone caused by DEX, and its incidence is secondary to primary osteoporosis [41]. Long-term high-dose injection of DEX leads to osteoblast apoptosis [42]. In this study, compared with untreated MC3T3-E1 cells, DEX-treated MC3T3-E1 cells inhibited osteogenesis, suggesting the successful establishment of DEX-induced osteoporosis cells. *CCNA1* was upregulated in DEX-induced osteoporosis cells.

Moreover, the knockdown of *CCNA1* significantly promoted osteogenesis in DEX-induced osteoporosis cells. Next, KEGG analysis suggested that *CCNA1* may suppress osteoblast differentiation via several signaling pathway including TGF-beta signaling pathway which plays an important role in maintaining bone homeostasis [43, 44]. Interestingly, BMP2, one of the evaluation marker of osteoblast differentiation, was reported to be a cytokine of TGF-beta family [23]. Subsequently, expression of two genes enriched in TGF-beta family was downregulated in DEX-induced osteoporosis cells. These results indicated that *CCNA1* may suppress TGF-beta signaling pathway in osteoporosis. Furthermore, the TGF-beta signaling pathway inhibitor LY2109761 significantly suppressed osteogenesis promoted by silenced *CCNA1* in DEX-induced osteoporosis cells, suggesting that *CCNA1* suppressed osteoblast differentiation by inactivating TGF-beta signaling pathway in osteoporosis.

The following limitations of this paper need to be further explored in the future. TGF-bata signaling pathway has been found to participate in CCNA1 modulating osteoblast differentiation process in vitro, the role of TGF-beta pathway should also be studied in vivo experiments in the future. Moreover, whether CCNA1 regulate the process of osteoporosis in a DEX-induced mouse model should also be studied.

## Conclusion

Taken together, CCNA1 inhibited osteoblast differentiation of osteoblasts via suppressing TGF-beta signaling pathway. Knockdown of CCNA1 may be an potential therapy for osteoporosis.

### Electronic supplementary material

Below is the link to the electronic supplementary material.

Supplementary Material 1

Supplementary Material 2

Supplementary Material 3

## Data Availability

The datasets used and/or analysed during the current study are available from the corresponding author on reasonable request (the direct link:https://www.ncbi.nlm.nih.gov/geo/query/acc.cgi?acc=GSE100609 accession number: GSE100609).

## References

[1] Lupsa BC, Insogna K. Bone Health and osteoporosis. Endocrin Metab Clin. [Journal Article; Review]. 2015 2015-09-01;44(3):517–30. 10.1016/j.ecl.2015.05.002 26316240

[2] Zhang L, Yin X, Wang J, Xu D, Wang Y, Yang J et al. Associations between VDR Gene Polymorphisms and osteoporosis risk and bone Mineral Density in Postmenopausal women: a systematic review and Meta-analysis. SCI REP-UK. [Journal Article; Meta-Analysis; Research Support, Non-U.S. Gov’t; Review; Systematic Review; Retracted Publication]. 2018 2018-01-17;8(1):981. 10.1038/s41598-017-18670-7 PMC5772668 29343720

[3] Black DM, Rosen CJ. Clinical Practice. Postmenopausal Osteoporosis. New Engl J Med. [Journal Article; Review]. 2016 2016-01-21;374(3):254 – 62. 10.1056/NEJMcp1513724 26789873

[4] Yin N, Zhu L, Ding L, Yuan J, Du L, Pan M et al. MiR-135-5p promotes osteoblast differentiation by targeting HIF1AN in MC3T3-E1 cells. Cell Mol Biol Lett. [Journal Article]. 2019 2019-01-20;24:51. 10.1186/s11658-019-0177-6 PMC6686269 31410089

[5] Rachner TD, Khosla S, Hofbauer LC. Osteoporosis: now and the future. Lancet. [Journal Article; Research Support, Non-U.S. Gov’t; Review]. 2011 2011-04-09;377(9773):1276–87. 10.1016/S0140-6736(10)62349-5 PMC3555696 21450337

[6] Florencio-Silva R, Sasso GRDS, Sasso-Cerri E, Simões MJ, Cerri PS, Lattanzi W. Biology of Bone tissue: structure, function, and factors that influence bone cells. Biomed Res INT. 2015 2015-01-01;2015:421717–46. 10.1155/2015/421746 PMC4515490 26247020

[7] Long Z, Dou P, Cai W, Mao M, Wu R. MiR-181a-5p promotes osteogenesis by targeting BMP3. Aging (Albany NY). [Journal Article; Research Support, Non-U.S. Gov’t]. 2023 2023-02-03;15(3):734–47. 10.18632/aging.204505 PMC9970307 36734882

[8] Radonova L, Pauerova T, Jansova D, Danadova J, Skultety M, Kubelka M et al. Cyclin A1 in Oocytes prevents chromosome segregation and anaphase entry. SCI REP-UK. [Journal Article; Research Support, Non-U.S. Gov’t]. 2020 2020-05-04;10(1):7455. 10.1038/s41598-020-64418-1 PMC7198627 32366979

[9] Chen Y, Zhao ZX, Huang F, Yuan XW, Deng L, Tang D. MicroRNA-1271 functions as a potential tumor suppressor in hepatitis B virus-associated hepatocellular carcinoma through the AMPK signaling pathway by binding to CCNA1. J Cell Physiol. [Journal Article; Research Support, Non-U.S. Gov’t]. 2019 2019-04-01;234(4):3555–69. 10.1002/jcp.26955 30565670

[10] Yang B, Miao S, Zhang LN, Sun HB, Xu ZN, Han CS. Correlation of CCNA1 promoter methylation with malignant tumors: a meta-analysis introduction. Biomed Res INT. [Journal Article; Meta-Analysis; Retracted Publication]. 2015 2015-01-20;2015:134027. 10.1155/2015/134027 PMC4310450 25654082

[11] Zhao R, Tao L, Qiu S, Shen L, Tian Y, Gong Z et al. Melatonin rescues glucocorticoid-induced inhibition of osteoblast differentiation in MC3T3-E1 cells via the PI3K/AKT and BMP/Smad signalling pathways. LIFE SCI. 2020 2020-01-01;257:118044. 10.1016/j.lfs.2020.118044 32622944

[12] Zheng Y, Xiao Y, Zhang D, Zhang S, Ouyang J, Li L et al. Geniposide ameliorated Dexamethasone-Induced cholesterol Accumulation in Osteoblasts by mediating the GLP-1R/ABCA1 Axis. Cells-Basel. [Journal Article; Research Support, Non-U.S. Gov’t]. 2021 2021-12-06;10(12). 10.3390/cells10123424 PMC8699812 34943934

[13] Xie B, Zeng Z, Liao S, Zhou C, Wu L, Xu D. Kaempferol ameliorates the inhibitory activity of Dexamethasone in the Osteogenesis of MC3T3-E1 cells by JNK and p38-MAPK pathways. Front Pharmacol. [Journal Article]. 2021 2021-01-20;12:739326. 10.3389/fphar.2021.739326 PMC8524096 34675808

[14] Qi M, Zhang L, Ma Y, Shuai Y, Li L, Luo K et al. Autophagy maintains the function of bone marrow mesenchymal stem cells to prevent Estrogen Deficiency-Induced osteoporosis. Theranostics [Journal Article]. 2017 2017-01-20;7(18):4498–516. 10.7150/thno.17949 PMC5695145 29158841

[15] Li J, Li X, Liu D, Hamamura K, Wan Q, Na S et al. eIF2α signaling regulates autophagy of osteoblasts and the development of osteoclasts in OVX mice. Cell Death Dis. 2019 2019-01-01;10(12):915–21. 10.1038/s41419-019-2159-z PMC6892793 31801950

[16] Chen S, Chen X, Geng Z, Su J. The horizon of bone organoid: a perspective on construction and application. Bioact Mater. 2022 2022-01-01;18:15–25. 10.1016/j.bioactmat.2022.01.048 PMC8961298 35387160

[17] Xue X, Hu Y, Wang S, Chen X, Jiang Y, Su J (2022). Fabrication of physical and chemical crosslinked hydrogels for bone tissue engineering. BIOACT MATER.

[18] Qiu Z, Li L, Huang Y, Shi K, Zhang L, Huang C et al. Puerarin specifically disrupts osteoclast activation via blocking integrin-β3 Pyk2/Src/Cbl signaling pathway. J Orthop Transl. 2022 2022-01-01;33:55–69. 10.1016/j.jot.2022.01.003 PMC8858883 35228997

[19] Lee WC, Guntur AR, Long F, Rosen CJ. Energy Metabolism of the osteoblast: implications for osteoporosis. ENDOCR REV. [Journal Article; Review; Research Support, N.I.H., Extramural]. 2017 2017-06-01;38(3):255–66. 10.1210/er.2017-00064 PMC5460680 28472361

[20] Greenblatt MB, Tsai JN, Wein MN. Bone turnover markers in the diagnosis and monitoring of metabolic bone disease. CLIN CHEM. [Journal Article; Review]. 2017 2017-02-01;63(2):464–74. 10.1373/clinchem.2016.259085 PMC5549920 27940448

[21] Jain S, Camacho P. Use of bone turnover markers in the management of osteoporosis. CURR OPIN ENDOCRINOL. [Journal Article; Review]. 2018 2018-12-01;25(6):366–72. 10.1097/MED.0000000000000446 30299435

[22] Cai N, Li C, Wang F. Silencing of LncRNA-ANCR promotes the Osteogenesis of osteoblast cells in postmenopausal osteoporosis via Targeting EZH2 and RUNX2. Yonsei Med J. [Journal Article]. 2019 2019-08-01;60(8):751–9. 10.3349/ymj.2019.60.8.751 PMC6660440 31347330

[23] Munoz J, Akhavan NS, Mullins AP, Arjmandi BH. Macrophage polarization and osteoporosis: a review. Nutrients. [Journal Article; Review]. 2020 2020-09-30;12(10). 10.3390/nu12102999 PMC7601854 33007863

[24] Filip R, Radzki RP, Bienko M. Novel insights into the relationship between nonalcoholic fatty liver disease and osteoporosis. Clin Interv Aging. [Journal Article; Review]. 2018 2018-01-20;13:1879–91. 10.2147/CIA.S170533 PMC6174895 30323574

[25] Chen X, Hua W, Huang X, Chen Y, Zhang J, Li G. Regulatory Role of RNA N(6)-Methyladenosine modification in Bone Biology and osteoporosis. Front Endocrinol. [Journal Article; Review]. 2019 2019-01-20;10:911. 10.3389/fendo.2019.00911 PMC6965011 31998240

[26] Cai P, Lu Y, Yin Z, Wang X, Zhou X, Li Z. Baicalein ameliorates osteoporosis via AKT/FOXO1 signaling. Aging (Albany, NY.). 2021 2021-01-01;13(13):17370–9. 10.18632/aging.203227 PMC8312461 34198266

[27] Orkin SH, Bauer DE. Emerging Genetic Therapy for Sickle Cell Disease. ANNU REV MED. [Journal Article; Research Support, N.I.H., Extramural; Research Support, Non-U.S. Gov’t; Review]. 2019 2019-01-27;70:257 – 71. 10.1146/annurev-med-041817-125507 30355263

[28] Brody H. Gene therapy. Nature. [Editorial; Introductory Journal Article]. 2018 2018-12-01;564(7735):S5. 10.1038/d41586-018-07639-9 30542191

[29] Lin C, Yu S, Jin R, Xiao Y, Pan M, Pei F et al. Circulating miR-338 cluster activities on osteoblast differentiation: potential diagnostic and therapeutic targets for postmenopausal osteoporosis. Theranostics. [Journal Article; Research Support, Non-U.S. Gov’t]. 2019 2019-01-20;9(13):3780–97. 10.7150/thno.34493 PMC6587346 31281513

[30] Raehtz S, Bierhalter H, Schoenherr D, Parameswaran N, McCabe LR. Estrogen Deficiency exacerbates type 1 Diabetes-Induced Bone TNF-alpha expression and osteoporosis in female mice. Endocrinology. [Journal Article; Research Support, Non-U.S. Gov’t]. 2017 2017-07-01;158(7):2086–101. 10.1210/en.2016-1821 PMC5505215 28419209

[31] Liu H, Zhao H, Lin H, Li Z, Xue H, Zhang Y et al. Relationship of COL9A1 and SOX9 genes with genetic susceptibility of postmenopausal osteoporosis. Calcified Tissue Int. [Journal Article]. 2020 2020-03-01;106(3):248–55. 10.1007/s00223-019-00629-7 31732751

[32] Kim YH, Jang WG, Oh SH, Kim JW, Lee MN, Song JH et al. Fenofibrate induces PPARalpha and BMP2 expression to stimulate osteoblast differentiation. Biochem Bioph Res Co. [Journal Article; Research Support, Non-U.S. Gov’t]. 2019 2019-12-03;520(2):459–65. 10.1016/j.bbrc.2019.10.048 31607484

[33] Hou Z, Wang Z, Tao Y, Bai J, Yu B, Shen J et al. KLF2 regulates osteoblast differentiation by targeting of Runx2. Lab Invest. [Journal Article; Research Support, Non-U.S. Gov’t]. 2019 2019-02-01;99(2):271–80. 10.1038/s41374-018-0149-x 30429507

[34] Chen Q, Shou P, Zheng C, Jiang M, Cao G, Yang Q et al. Fate decision of mesenchymal stem cells: adipocytes or osteoblasts? Cell Death Differ. [Journal Article; Review]. 2016 2016-07-01;23(7):1128–39. 10.1038/cdd.2015.168 PMC4946886 26868907

[35] Birmingham E, Niebur GL, McHugh PE, Shaw G, Barry FP, McNamara LM. Osteogenic differentiation of mesenchymal stem cells is regulated by osteocyte and osteoblast cells in a simplified bone niche. Eur Cells Mater. [Comparative study; Journal Article; Research Support, Non-U.S. Gov’t]. 2012 2012-01-12;23:13–27. 10.22203/ecm.v023a02 22241610

[36] Ciubean AD, Ungur RA, Irsay L, Ciortea VM, Borda IM, Dogaru GB et al. Polymorphisms of FDPS, LRP5, SOST and VKORC1 genes and their relation with osteoporosis in postmenopausal Romanian women. Plos One. [Journal Article; Observational Study]. 2019 2019-01-20;14(11):e225776. 10.1371/journal.pone.0225776 PMC6880991 31774873

[37] Weivoda MM, Youssef SJ, Oursler MJ. Sclerostin expression and functions beyond the osteocyte. BONE. [Journal Article; Review; Research Support, Non-U.S. Gov’t; Research Support, N.I.H., Extramural]. 2017 2017-03-01;96:45–50. 10.1016/j.bone.2016.11.024 PMC5328839 27888056

[38] Bonnet N, Bourgoin L, Biver E, Douni E, Ferrari S. RANKL inhibition improves muscle strength and insulin sensitivity and restores bone mass. J Clin Invest. [Journal Article; Research Support, Non-U.S. Gov’t]. 2019 2019-05-23;129(8):3214–23. 10.1172/JCI125915 PMC6668701 31120440

[39] Lv F, Cai X, Yang W, Gao L, Chen L, Wu J et al. Denosumab or romosozumab therapy and risk of cardiovascular events in patients with primary osteoporosis: systematic review and meta- analysis. BONE. [Journal Article; Meta-Analysis; Research Support, Non-U.S. Gov’t; Systematic Review]. 2020 2020-01-01;130:115121. 10.1016/j.bone.2019.115121 31678488

[40] Ruaro B, Casabella A, Paolino S, Pizzorni C, Ghio M, Seriolo C et al. Dickkopf-1 (Dkk-1) serum levels in systemic sclerosis and rheumatoid arthritis patients: correlation with the trabecular bone score (TBS). Clin Rheumatol. [Journal Article]. 2018 2018-11-01;37(11):3057–62. 10.1007/s10067-018-4322-9 30291470

[41] Qin Z, Li S, Zhang X, Liu G, Gu M, Zhang N et al. Combination therapy of Wuweizi (Schisandrae Chinensis Fructus) and dexamethasone alleviated Dexamethasone-Induced glucocorticoid osteoporosis in rats with idiopathic pulmonary fibrosis. Biomed Res Int. [Journal Article]. 2020 2020-01-20;2020:6301697. 10.1155/2020/6301697 PMC7115146 32280693

[42] Han D, Gu X, Gao J, Wang Z, Liu G, Barkema HW et al. Chlorogenic acid promotes the Nrf2/HO-1 anti-oxidative pathway by activating p21(Waf1/Cip1) to resist dexamethasone-induced apoptosis in osteoblastic cells. Free Radical Bio Med. [Journal Article; Research Support, Non-U.S. Gov’t]. 2019 2019-06-01;137:1–12. 10.1016/j.freeradbiomed.2019.04.014 31004750

[43] Morikawa M, Derynck R, Miyazono K. TGF-beta and the TGF-beta family: context-dependent roles in cell and tissue physiology. CSH Perspect Biol. [Journal Article; Review]. 2016 2016-05-02;8(5). 10.1101/cshperspect.a021873 PMC4852809 27141051

[44] Clark DA, Coker R. Transforming growth factor-beta (TGF-beta). INT J Biochem Cell B. [Journal Article; Review]. 1998 1998-03-01;30(3):293–8. 10.1016/s1357-2725(97)00128-3 9611771

