# Supplementary material for: Cyclin A1 (CCNA1) inhibits osteoporosis by suppressing transforming growth factor-beta (TGF-beta) pathway in osteoblasts
Source: BMC Musculoskelet Disord. 2024 Mar 7;25:206. doi: 10.1186/s12891-024-07303-6 (PMC10919014; doi:10.1186/s12891-024-07303-6)
Supplement: Supplementary file 3 — Supplementary Material 3 [file 12891_2024_7303_MOESM3_ESM.pdf]

KEGG Database Project
Kanehisa Laboratories
www.kanehisa.jp

Ref: 232300

Permission is granted to BMC Musculoskeletal Disorders to publish under the CC BY 4.0 open access license the following KEGG pathway map image in the article "Cyclin A1 (CCNA1) inhibits osteoporosis by suppressing transforming growth factor-beta (TGF-beta) pathway in osteoblasts" written by Qinglei Wang and colleagues:

- TGF-beta signaling pathway (map04350)

subject to the condition that the original source is acknowledged by citing at least one KEGG paper.

Permission granted: Naomi Takahashi

Naomi Takahashi, Kanehisa Laboratories

Date: 30 November 2023

Copyright holder: Kanehisa Laboratories
