# Supplementary material for: Cyclin A1 (CCNA1) inhibits osteoporosis by suppressing transforming growth factor-beta (TGF-beta) pathway in osteoblasts
Source: BMC Musculoskelet Disord. 2024 Mar 7;25:206. doi: 10.1186/s12891-024-07303-6 (PMC10919014; doi:10.1186/s12891-024-07303-6)
Supplement: Supplementary file 1 — Supplementary Material 1 [file 12891_2024_7303_MOESM1_ESM.doc]

ARS:

F2

| Control | DEX |
| --- | --- |
|  |  |

F3

| si-NC | si-CCNA1 |
| --- | --- |
|  |  |

F5

| si-NC | si-CCNA1 | Si-CCNA1+LY2109761 |
| --- | --- | --- |
|  |  |  |

ALP:

F2

| Control | DEX |
| --- | --- |
|  |  |

F3

| si-NC | si-CCNA1 |
| --- | --- |
|  |  |

F5

| si-NC | si-CCNA1 | Si-CCNA1+LY2109761 |
| --- | --- | --- |
|  |  |  |

WB

F2D

| ALP |  |
| --- | --- |
| OCN |  |
| OPN |  |
| Osterix |  |
| BMP2 |  |
| GAPDH |  |

F3E

| ALP |  |
| --- | --- |
| OCN |  |
| OPN |  |
| Osterix |  |
| BMP2 |  |
| GAPDH |  |

F4C

| IFNB1 |  |
| --- | --- |
| PARD6 |  |
| p-SMAD2 |  |
| SMAD2 |  |
| GAPDH |  |

F5A

| p-SMAD2 |  |
| --- | --- |
| SMAD2 |  |
| p-SMAD3 |  |
| SMAD3 |  |
| GAPDH |  |

F5E

| ALP |  |
| --- | --- |
| OCN |  |
| OPN |  |
| Osterix |  |
| BMP2 |  |
| GAPDH |  |

CT图

| Sham | OVX | OVX+LV-sh-NC | OVX+LV-sh-CCNA1 |
| --- | --- | --- | --- |
|  |  |  |  |

HE染色

| Sham | OVX | OVX+LV-sh-NC | OVX+LV-sh-CCNA1 |
| --- | --- | --- | --- |
|  |  |  |  |
